# Age Patterns of HSV-2 Incidence and Prevalence in Two Ugandan Communities: A Catalytic Incidence Model Applied to Population-Based Seroprevalence Data

**DOI:** 10.1093/infdis/jiad113

**Published:** 2023-04-20

**Authors:** Lilith K Whittles, Ronald M Galiwango, Josephine Mpagazi, Aaron A R Tobian, Robert Ssekubugu, Jade Jackson, Austin D Peer, Caitlin Kennedy, Margaret Nakalanzi, Anthony Ndyanabo, Godfrey Kigozi, Larry W Chang, David Serwadda, Yukari C Manabe, Charlotte A Gaydos, Oliver Laeyendecker, Thomas C Quinn, Steven J Reynolds, Joseph Kagaayi, Jeffrey W Eaton, M Kate Grabowski

**Affiliations:** Department of Infectious Disease Epidemiology, School of Public Health, Imperial College London, London, United Kingdom; Medical Research Council Centre for Global Infectious Disease Analysis, and NIHR Health Protection Research Unit in Modelling and Health Economics, School of Public Health, Imperial College London, London, United Kingdom; Rakai Health Sciences Program, Kalisizo, Uganda; Rakai Health Sciences Program, Kalisizo, Uganda; Department of Pathology, Johns Hopkins School of Medicine, Baltimore, Maryland, USA; Department of Epidemiology, Johns Hopkins Bloomberg School of Public Health, Baltimore, Maryland, USA; Rakai Health Sciences Program, Kalisizo, Uganda; Department of Pathology, Johns Hopkins School of Medicine, Baltimore, Maryland, USA; Johns Hopkins School of Medicine, Baltimore, Maryland, USA; Rakai Health Sciences Program, Kalisizo, Uganda; Department of International Health, Johns Hopkins Bloomberg School of Public Health, Baltimore, Maryland, USA; Rakai Health Sciences Program, Kalisizo, Uganda; Rakai Health Sciences Program, Kalisizo, Uganda; Rakai Health Sciences Program, Kalisizo, Uganda; Rakai Health Sciences Program, Kalisizo, Uganda; Department of Epidemiology, Johns Hopkins Bloomberg School of Public Health, Baltimore, Maryland, USA; Department of International Health, Johns Hopkins Bloomberg School of Public Health, Baltimore, Maryland, USA; Department of Medicine, Johns Hopkins University, Baltimore, Maryland, USA; Rakai Health Sciences Program, Kalisizo, Uganda; Department of Disease Control and Environmental Health, Makerere University School of Public Health, Kampala, Uganda; Department of Medicine, Johns Hopkins University, Baltimore, Maryland, USA; Department of Medicine, Johns Hopkins University, Baltimore, Maryland, USA; Department of Epidemiology, Johns Hopkins Bloomberg School of Public Health, Baltimore, Maryland, USA; Department of Medicine, Johns Hopkins University, Baltimore, Maryland, USA; Laboratory of Immunoregulation, Division of Intramural Research, National Institute for Allergy and Infectious Diseases, National Institutes of Health, Bethesda, Maryland, USA; Rakai Health Sciences Program, Kalisizo, Uganda; Department of Epidemiology, Johns Hopkins Bloomberg School of Public Health, Baltimore, Maryland, USA; Department of Medicine, Johns Hopkins University, Baltimore, Maryland, USA; Laboratory of Immunoregulation, Division of Intramural Research, National Institute for Allergy and Infectious Diseases, National Institutes of Health, Bethesda, Maryland, USA; Rakai Health Sciences Program, Kalisizo, Uganda; Department of Epidemiology, Johns Hopkins Bloomberg School of Public Health, Baltimore, Maryland, USA; Department of Medicine, Johns Hopkins University, Baltimore, Maryland, USA; Laboratory of Immunoregulation, Division of Intramural Research, National Institute for Allergy and Infectious Diseases, National Institutes of Health, Bethesda, Maryland, USA; Department of Epidemiology, Johns Hopkins Bloomberg School of Public Health, Baltimore, Maryland, USA; Department of Disease Control and Environmental Health, Makerere University School of Public Health, Kampala, Uganda; Medical Research Council Centre for Global Infectious Disease Analysis, and NIHR Health Protection Research Unit in Modelling and Health Economics, School of Public Health, Imperial College London, London, United Kingdom; Rakai Health Sciences Program, Kalisizo, Uganda; Department of Pathology, Johns Hopkins School of Medicine, Baltimore, Maryland, USA; Department of Epidemiology, Johns Hopkins Bloomberg School of Public Health, Baltimore, Maryland, USA

**Keywords:** HSV-2, catalytic model, incidence, population-based study, prevalence

## Abstract

**Background:**

Herpes simplex virus type 2 (HSV-2) is an incurable sexually transmitted infection associated with increased risk of acquiring and transmitting human immunodeficiency virus (HIV). HSV-2 is highly prevalent in sub-Saharan Africa, but population-level estimates of incidence are sparse.

**Methods:**

We measured HSV-2 prevalence from cross-sectional serological data among adults aged 18–49 years in 2 south-central Uganda communities (fishing, inland). We identified risk factors for seropositivity, then inferred age patterns of HSV-2 with a Bayesian catalytic model.

**Results:**

HSV-2 prevalence was 53.6% (n = 975/1819; 95% confidence interval, 51.3%–55.9%). Prevalence increased with age, was higher in the fishing community, and among women, reaching 93.6% (95% credible interval, 90.2%–96.6%) by age 49 years. Factors associated with HSV-2 seropositivity included more lifetime sexual partners, HIV positive status, and lower education. HSV-2 incidence peakied at age 18 years for women and 19–20 years for men. HIV prevalence was up to 10-fold higher in HSV-2–positive individuals.

**Conclusions:**

HSV-2 prevalence and incidence were extremely high, with most infections occurring in late adolescence. Interventions against HSV-2, such as future vaccines or therapeutics, must target young populations. Remarkably higher HIV prevalence among HSV-2–positive individuals underscores this population as a priority for HIV prevention.

## Teaser

Prevalence of HSV-2 is high in south-central Uganda, particularly among women, fishing communities, and people with HIV. HSV-2 acquisition peaks in late adolescence. Remarkably higher HIV prevalence among people with HSV-2 makes this group a priority for HIV prevention.

Herpes simplex virus type 2 (HSV-2) is a sexually transmitted infection (STI) that is prevalent in half a billion people worldwide [1]. Burden is highest in sub-Saharan Africa, where population prevalence is estimated at 37% (95% CI, 35%–40%) [2]. Once acquired, infection is lifelong and is the leading cause of genital ulcer disease [3].

HSV-2 infection is associated with 3-fold higher risk of acquiring human immunodeficiency virus (HIV), and 5 times higher among those recently HSV-2 infected [4]. Increased HIV risk is due to a combination of biological factors and shared underlying sexual risk factors. HSV-2 and HIV acquisition share common determinants of infection including position within sexual networks and behavioral factors, such as condom use and frequency of partner change [5, 6]. Numerous biological hypotheses for how HSV-2 increases HIV acquisition include increased HSV-2–induced genital ulcers and recruitment of CD4^+^ T cells and dendritic cells to the mucosa that express CCR5 [7–9]. The relationship between HSV-2 and risk of HIV infection has important consequences in areas with cocirculating epidemics, and has been estimated to account for more than a third of new HIV infections in the World Health Organization (WHO) African region each year [10, 11].

The increased risk of HIV infection associated with acute HSV-2 infection underlies the importance of understanding patterns of HSV-2 incidence at the population level to better target HIV prevention measures towards the most at-risk groups. Of particular interest is quantifying how HSV-2 incidence varies with respect to age, sex, and location, with a view towards ameliorating HSV-2–related morbidity and transmission, including via therapeutics or future vaccines. Despite the importance of HSV-2 and HIV prevention efforts in areas of high burden, serological studies of HSV-2 incidence are generally lacking, in part due to the substantial administrative and financial cost of population-based studies. However, the lifelong nature of HSV-2 infection means seroprevalence data can be leveraged to infer incidence from how prevalence changes with age [12].

In this study, we describe determinants of HSV-2 infection among men and women in 2 distinct communities in Rakai, south-central Uganda. The region reported the first cases of HIV in East Africa [13], and continues to have the highest burden of HIV nationally [14]. The HSV-2 seroprevalence study was embedded within the long-running Rakai Community Cohort Study (RCCS) [15]. We analyze patterns of HIV and HSV-2 infection and fit a parametric catalytic model of infection to age-specific seroprevalence to estimate age patterns of HSV-2 incidence.

## METHODS

### Data

We analyzed data from the Sexually Transmitted Infection Prevalence Study (STIPS), a community-based cross-sectional study of STI prevalence among adults in south-central Uganda undertaken between May and October 2019. The study was nested within RCCS, a long-running population-based cohort conducted by the Rakai Health Sciences Program (RHSP) [16]. The RHSP refers participants for HIV treatment and prevention services, including antiretroviral therapy, voluntary medical male circumcision, and preexposure prophylaxis (PrEP), which has been rolled out to high-risk populations since 2017 [17]. Detailed study methods for STIPS have been described previously [18]. Briefly, STIPS was conducted among consenting RCCS participants aged 18–49 years from 2 communities: a Lake Victoria fishing community, and an inland community comprising a semiurban trading center and surrounding agrarian villages. The communities were chosen to reflect the substantial differences in HIV prevalence and sexual behaviors present in the region, based on geographic diversity, population size, and enrolment timeline within the wider RCCS survey [16]. RCCS eligibility is based on household census of people resident for ≥6 months in inland communities and ≥1 month with intention to stay longer in fishing communities. While the RCCS enrolls participants aged 15 and above, resource limitations did not allow the inclusion of people younger than 18 years in STIPS. Enrolled participants were surveyed about STI symptoms and treatment seeking. Circumcision status for male participants was self-reported, then clinically confirmed. History of PrEP usage was self-reported. Participants provided genital swabs; penile swabs were collected by clinicians while vaginal swabs were self-administered, which has been shown to be more acceptable to women without impacting test sensitivity [19].

Following standard RCCS methodology, sera from peripheral blood samples were tested for HSV-2 using the Kalon ELISA (Kalon Biological Ltd) with an index cutoff value of 1.5, which gave optimal test sensitivity and specificity in a Ugandan population in previous validation [20]. All HSV-2 tests were performed at the RHSP central laboratory in Kalisizo, Uganda. HIV serological testing used a field-validated parallel 3-test rapid algorithm [21]. Syphilis seropositivity, defined by treponemal antibody status, was determined using the SD Bioline Syphilis 3.0 (SD Biostandard Diagnostics, Private Limited). Syphilis titers were determined at the RHSP central laboratory for all participants with positive serology, using the rapid plasma reagin (RPR) nontreponemal test (Cypress Diagnostics). High-titer syphilis infection was defined as RPR titer ≥1:8. Genital swabs were tested for trichomonas, chlamydia, and gonorrhea. Trichomonas tests were performed using the OSOM Trichomonas Rapid Test (Sekisui). Chlamydia and gonorrhea tests were performed at the RHSP central laboratory, using the Abbott m2000 RealTime CT/NG assay.

HIV, syphilis, and trichomonas point-of-care test results were returned by on-site counsellors. Participants who tested positive and their partners were treated in line with Uganda Clinical Guidelines and United States Centers for Disease Control and Prevention (CDC) guidelines for treatment of STIs [22, 23]. Partners were identified via passive referral. Participants diagnosed with chlamydia and gonorrhea were recontacted and provided with counselling, etiologic treatment, and referrals for their sexual partners, who were invited to obtain presumptive STI treatment. Participants who tested negative but reported STI symptoms (genital discharge or lesions) were nevertheless offered antibiotic treatment in line with both Ugandan national guidelines for syndromic management of STIs [23].

### Ethics

STIPS was approved by Uganda Virus Research Institute Research Ethics Committee (GC/127/19/03/709), the Johns Hopkins School of Medicine Institutional Review Board (IRB00204691), and was registered with the Ugandan National Council for Science and Technology (HS 364 ES) [18]. All study participants provided written informed consent for STIPS in addition to the RCCS. The consent for STIPS was read to all participants by a research assistant; where participants were illiterate, a literate community member (of the participant's choice but typically the training community health worker) was present to witness the consent process, with consent confirmed via the participant's thumbprint in the place of a signature. All participants regardless of their literacy received a copy of the signed consent form for future reference.

### Statistical Methods

We compared demographic and behavioral characteristics of STIPS participants in inland and fishing communities using χ^2^ tests. We estimated HSV-2 seroprevalence in men and women, and used modified Poisson regression [24] to estimate the association between HSV-2 infection and risk factors, specifically: community type (inland/fishing), age group, marital status, educational level, number of sex partners in the last year, lifetime number of sex partners, HIV serostatus, HIV viral load suppression status (defined as <1000 copies/mL, as per WHO guidelines [25]), history of PrEP usage, male circumcision status, and infection with a curable STI (trichomonas, chlamydia, gonorrhea, and syphilis). We measured all associations using unadjusted and adjusted prevalence risk ratios (PRR, adj PRR) with 95% confidence intervals (CI). Adjusted models controlled for age group, community type, marital status, educational level, and HIV serostatus.

### Model Structure and Calibration

To infer HSV-2 incidence by age from seroprevalence data, we fitted a catalytic model to HSV-2 test data separately for men and women in inland and fishing communities [12]. We represented prevalence at each age using the cumulative distribution function of a shifted generalized gamma distribution [26, 27]. We implemented the model in a Bayesian framework using the probabilistic programming language Stan [28] and used an adaptive Hamiltonian Monte Carlo no U-turn sampler to estimate model parameters for each sex and location separately. Because infection with HSV-2 is lifelong, modelled incidence rates are given by the probability density function specified by the fitted model parameters. Full details of the model structure and calibration can be found in the Supplementary Material.

## RESULTS

### Study Population Characteristics

Of the 2583 RCCS-eligible individuals aged 18–49 years, 73% (1884) were present in the community at the time of the census, of whom 97% (1825) agreed to participate in STIPS. The study population comprised 522 women and 397 men in the inland community and 443 women and 463 men in the fishing community (Table 1). Invited individuals who did not participate in the STIPS study were significantly more likely to be men, younger aged, reside in the fishing community, have unknown HIV serostatus, or be recent in-migrants [18]. In both communities, men were less likely to be HIV seropositive than women but reported more sexual partners both in the last year and over their lifetime. More than half of men were circumcised: 52% inland and 57% in the fishing community. Very few participants in the inland community had ever taken PrEP (1/522 women, and 3/397 men). PrEP usage was relatively higher in the fishing community, comprising only 8.5% of women and 6.5% of men. Data on curable STI positivity have been previously reported by Grabowski et al [18].

**Table 1.** Characteristics of Study Population by Sex and Community Type

	Inland (n = 919)			Fishing (n = 906)		
Characteristic	Women (n = 522)	Men (n = 397)	*P* Value	Women (n = 443)	Men (n = 463)	*P* Value
Age, y, median (IQR)	32 (24–39)	30 (23–39)		31 (26–38)	32 (26–30)	
Marital status			< .0001			< .0001
Never married	67 (13)	102 (26)		22 (5.0)	71 (15)	
Currently married	350 (67)	255 (64)		306 (69)	289 (62)	
Previously married	105 (20)	40 (10)		115(26)	103 (22)	
Educational status			.15			.04
None	25 (5.6)	11 (2.8)		56 (13)	41 (8.9)	
Primary	292 (56)	245 (62)		277 (63)	330 (71)	
Secondary	145(28)	106 (27)		83 (19)	71 (15)	
Tertiary	60 (11)	35 (8.8)		27 (6.1)	21 (4.5)	
Sex partners in the last year			< .0001			< .0001
0	75 (14)	42 (10.6)		37 (8.4)	25 (5.4)	
1	406 (78)	165 (42)		332 (75)	153 (33)	
2	32 (6.1)	117 (29)		54 (12)	136 (29)	
3	8 (1.5)	44 (11)		11 (2.5)	82 (18)	
≥4	1 (0.2)	29 (7.3)		9 (2.0)	67 (15)	
Lifetime sex partners			< .0001			< .0001
0	20 (3.8)	22 (5.5)		4 (0.9)	10 (2.2)	
1	99 (19)	14 (3.5)		34 (7.7)	9 (1.9)	
2	155 (30)	29 (7.3)		60 (14)	10 (2.2)	
3	132 (25)	41 (10)		108 (24)	27 (5.8)	
≥4	116 (22)	291 (73)		237 (53)	407 (88)	
HIV serostatus			.02			< .0001
HIV seronegative	436 (84)	354 (89)		236 (53)	309 (67)	
HIV seropositive	86 (16)	43 (11)		297 (47)	154 (33)	
HIV suppression status^a^			.22			.36
≤1000 copies/mL	81 (94)	37 (86)		190 (92)	136 (88)	
>1000 copies/mL	5 (6)	6 (14)		17 (8)	18 (12)	
Current or prior history of PrEP use^b^			.48			.58
No	434 (99)	351 (99)		216 (92)	289 (94)	
Yes	1 (0.2)	3 (0.8)		19 (8.1)	20 (6.5)	
Male circumcision status^c^						
Uncircumcised	…	189 (48)		…	199 (43)	
Circumcised	…	208 (52)		…	264 (57)	

Total number of participants and percentage (%) shown unless otherwise noted.

Abbreviations: HIV, human immunodeficiency virus; IQR, interquartile range; PrEP, preexposure prophylaxis.

Analysis restricted to HIV-seropositive participants.

Analysis restricted to HIV-seronegative participants; 2 participants had missing data.

Analysis restricted to male study participants.

### Risk Factors Associated With HSV-2 Seropositivity

Prevalence of HSV-2 in the study population was 53.6% (95% CI, 51.3%–55.9%). Prevalence was higher in women (63.1%; 95% CI, 60.0%–66.2%) than in men (43.0%; 95% CI, 39.6%–46.3%), and higher in the fishing community than inland (64.4%; 95% CI, 61.2%–67.5% vs 43.0%; 95% CI, 39.7%–46.2%). Women in the fishing community were 1.23 (95% CI, 1.11–1.36) times more likely to have HSV-2 than women inland (Table 2). Among men, crude prevalence was also higher in the fishing community (51% vs 34%), but the difference was not significant after adjusting for confounders. The seroprevalence of HSV-2 increased rapidly with age. In the oldest age group (45–49 years), women were 1.90 (95% CI, 1.55–2.32) times and men were 2.26 (95% CI, 1.49–3.42) times more likely to be seropositive than those aged 15–24 years.

**Table 2.** Risk Factors Associated With HSV-2 seropositivity

	Women (n = 960)^a^			Men (n = 859)^a^		
Risk Factor	No. HSV-2 Positive/Total (%)	PRR (95% CI)	adjPRR (95% CI)	No. HSV-2 Positive/Total (%)	PRR (95% CI)	adjPRR (95% CI)
Community type						
Inland	260/518 (50)	Ref.	Ref.	133/397 (34)	Ref.	Ref.
Fishing	346/442 (78)	**1.56 (1.41–1.72)**	**1.23 (1.11–1.36)**	236/462 (51)	**1.52 (1.29–1.80)**	1.11 (.94–1.32)
Age group, y						
15–24	77/235 (33)	Ref.	Ref.	30/203 (15)	Ref.	Ref.
25–29	116/183 (63)	**1.94 (1.56–2.40)**	**1.44 (1.19–1.75)**	59/164 (36)	**2.43 (1.65–3.56)**	**1.53 (1.03–2.29)**
30–34	122/164 (84)	**2.27 (1.85–2.78)**	**1.55 (1.29–1.88)**	72/140 (51)	**3.48 (2.41–5.03)**	**1.94 (1.32–2.86)**
35–39	129/174 (84)	**2.26 (1.85–2.77)**	**1.65 (1.37–2.00)**	88/156 (56)	**3.82 (2.67–5.46)**	**2.15 (1.46–3.15)**
40–44	99/131 (76)	**2.31 (1.87–2.84)**	**1.59 (1.31–1.94)**	76/122 (62)	**4.21 (2.95–6.03)**	**2.25 (1.52–3.33)**
45–49	63/73 (86)	**2.63 (2.15–3.23)**	**1.90 (1.55–2.32)**	44/74 (60)	**4.02 (2.75–5.89)**	**2.26 (1.49–3.42)**
Marital status						
Never married	22/88 (25)	**0.40 (.28–.58)**	0.75 (.56–1.00)	25/173 (15)	**0.30 (.20–.40)**	0.75 (.51–1.12)
Currently married	406/652 (62)	Ref.	Ref.	270/543 (50)	Ref.	Ref.
Previously married	178/220 (81)	**1.30 (1.19–1.42)**	1.04 (.95–1.14)	74/143 (52)	1.04 (.88–1.25)	1.02 (.85–1.22)
Educational status						
None	66/81 (82)	**1.18 (1.05–1.33)**	1.01 (.90–1.13)	32/52 (62)	**1.33 (1.06–1.68)**	0.98 (.78–1.24)
Primary	39/565 (69)	Ref.	Ref.	265/574 (46)	Ref.	Ref.
Secondary	108/227 (48)	**0.69 (.59–.80)**	0.91 (.80–1.03)	59/177 (33)	**0.72 (.58–.91)**	0.82 (.66–1.01)
Tertiary	42/87 (48)	**0.70 (.56–.88)**	0.91 (.74–1.12)	13/56 (23)	**0.50 (.31–.82)**	**0.51 (.32–.82)**
Sex partners in the last year						
0	64/112 (57)	0.92 (.77–1.09)	0.98 (.86–1.11)	9/67 (13)	**0.30 (.16–.56)**	0.80 (.48–1.33)
1	457/733 (62)	Ref.	Ref.	143/318 (45)	Ref.	…
2	61/86 (71)	1.14 (.98–1.32)	1.02 (.89–1.17)	110/252 (44)	0.98 (.81–1.17)	0.99 (.83–1.17)
3	17/19 (90)	**1.44 (1.22–1.69)**	**1.23 (1.04–1.46)**	67/126 (53)	1.18 (.96–1.45)	**1.22 (1.00–1.47)**
≥4	7/10 (70)	1.12 (.75–1.69)	0.76 (.57–1.01)	40/96 (42)	0.93 (.71–1.21)	0.92 (.71–1.19)
Lifetime sex partners						
0	0/24 (0)	…	…	0/32 (0)	…	…
1	39/130 (30)	Ref.	Ref.	1/23 (4.3)	Ref.	Ref.
2	115/214 (54)	**1.79 (1.34–2.39)**	**1.63 (1.24–2.13)**	10/38 (26.3)	6.05 (.83–44.3)	4.13 (.57–30.10)
3	167/239 (70)	**2.32 (1.77–3.07)**	**1.79 (1.38–2.32)**	34/68 (35.3)	**8.12 (1.16–56.7)**	4.04 (.58–28.03)
≥4	285/353 (81)	**2.69 (2.06–3.52)**	**1.81 (1.39–2.35)**	334/698 (47.9)	**11.0 (1.62–75.0)**	4.33 (.63–29.76)
HIV serostatus						
HIV seronegative	332/667 (50)	Ref.	Ref.	219/662 (33)	Ref.	Ref.
HIV seropositive	274/293 (94)	**1.88 (1.73–2.04)**	**1.39 (1.28–1.50)**	150/197 (76)	**2.30 (2.01–2.63)**	**1.58 (1.37–1.83)**
HIV suppression status^b^						
≤1000 copies/mL	255/271 (94)	Ref	Ref	133/173 (77)	Ref.	Ref
>1000 copies/mL	19/22 (86)	0.92 (.78–1.09)	0.91 (.76–1.10)	17/24 (71)	0.92 (.70–1.21)	0.98 (.76–1.27)
Current or prior history of PrEP use^c^						
No	316/645 (49)	Ref	Ref	208/639 (33)	Ref.	Ref
Yes	15/20 (75)	**1.53 (1.17–2.00)**	1.00 (.77–1.29)	11/23 (48)	1.47 (.95–2.28)	1.08 (.67–1.75)
Male circumcision status						
Uncircumcised	…	…	…	193/388 (63)	Ref.	Ref
Circumcised	…	…	…	176/471 (37)	**0.75 (.65–.88)**	0.90 (.78–1.04)

Bold text shows statistically significant results.

Abbreviations: adjPRR, adjusted prevalence risk ratio models adjusted for age, community type, marital status, educational level, and HIV serostatus; CI, confidence interval; HIV, human immunodeficiency virus; HSV, herpes simplex virus; PrEP, preexposure prophylaxis; PRR, prevalence risk ratio; Ref., reference.

There were 6 study participants with missing HSV-2 results (n = 5 women; n = 1 man).

Analysis restricted to HIV-seropositive participants.

Analysis restricted to HIV-negative participants.

Marital status was not significantly associated with HSV-2 prevalence. However, reporting more lifetime sexual partners was associated with higher seroprevalence in women: 81% of women reporting 4 or more partners had HSV-2, compared to 30% of women reporting only 1 partner (adjPRR 1.81; 95% CI, 1.39–2.35). Men with tertiary education had much lower prevalence of HSV-2: 23% compared to 46% among men with primary education only (adjPRR 0.51; 95% CI, .32–.82).

Individuals with HIV were significantly more likely to be HSV-2 seropositive, with a pronounced difference in both men (76% vs 33%; adjPRR = 1.58; 95% CI, 1.37–1.83) and women (94% vs 50%; adjPRR = 1.39; 95% CI, 1.28–1.50). HIV load suppression status was not significantly associated with HSV-2 prevalence. Circumcised men had lower HSV-2 seropositivity (37% vs 63%; PRR 0.75; 95% CI, .65–.88); however, the difference was smaller and not significant after adjusting for age and other confounders (adjPRR 0.90, 95% CI, .78–1.04). For women, history of PrEP use was associated with risk of HSV-2, although the association was not significant after adjusting for confounders that may reflect underlying sexual risk.

### Associations Between Curable STIs and HSV-2 Seroprevalence by Sex

Adjusting for age, community type, HIV status, educational level, and marital status, neither gonorrhea, chlamydia, nor high-titer syphilis infection (nontreponemal antibody titer ≥ 1:8) were significantly associated with HSV-2 infection (Supplementary Table 1). Trichomonas was associated with HSV-2 infection in women (adjPRR 1.22; 95% CI, 1.12–1.34), but not in men (adjPRR 0.95; 95% CI, .71–1.28), whereas syphilis treponemal seropositivity (regardless of titer) was associated with HSV-2 infection in men (adjPRR 1.26; 95% CI, 1.08–1.46), but not in women (adjPRR 1.05; 95% CI, .96–1.15).

### HIV Prevalence by HSV-2 Serostatus

**Figure 1.**
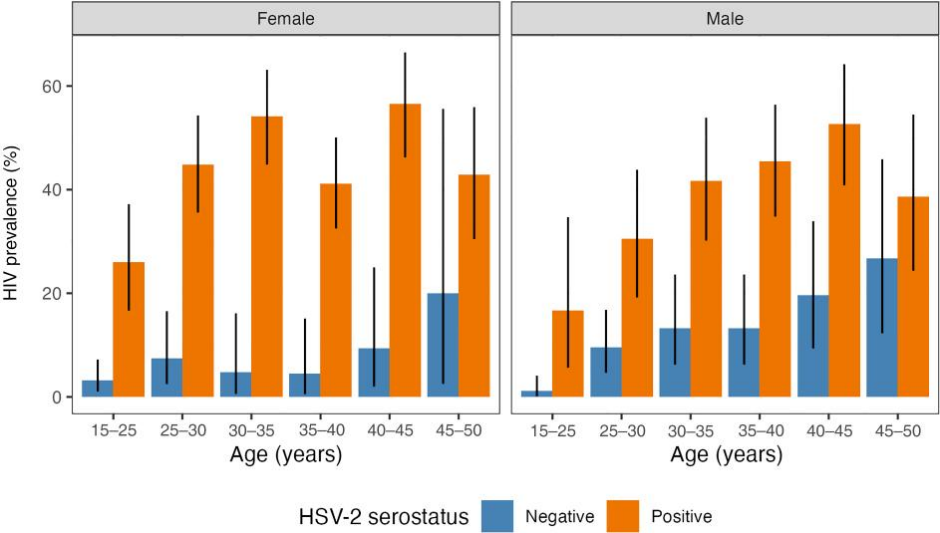
Comparison of human immunodeficiency virus (HIV) prevalence by age stratified by herpes simplex virus-2 (HSV-2) serostatus and sex. Bars show HIV prevalence in each age group, with lines showing 95% binomial confidence intervals.

HIV prevalence was significantly higher among men and women with HSV-2 across all age groups. Among women, 54.1% (95% CI, 44.8%–63.2%) of HSV-2–seropositive women aged 30–35 years were also HIV positive, compared to only 4.8% (95% CI, .6%–16.2%) of HSV-2–seronegative women (Figure 1). Among men, 41.7% (95% CI, 30.2%–53.9%) of HSV-2–seropositive men aged 30–35 years were also HIV positive, compared to only 13.2% (95% CI, 6.2%–23.6%) of HSV-2–seronegative men.

### Catalytic Model, HSV-2 Seroprevalence and Incidence

**Figure 2.**
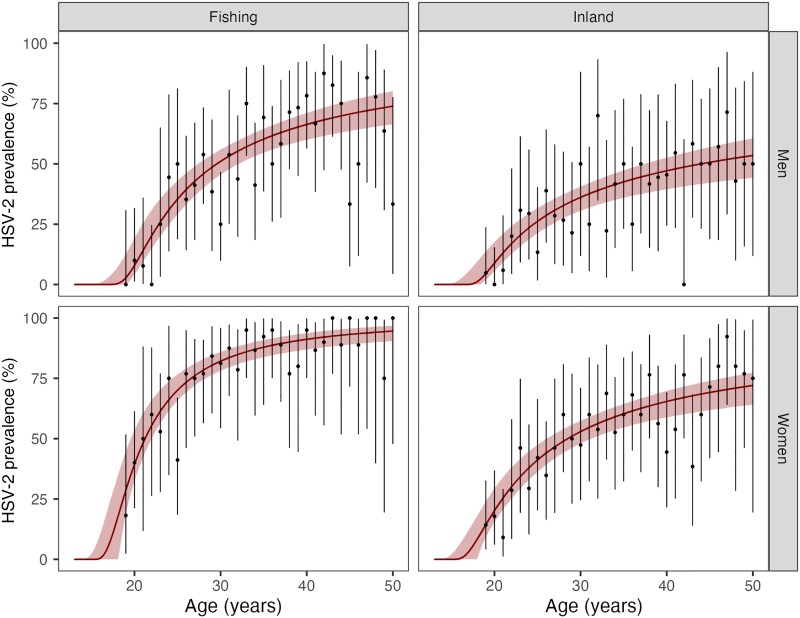
Herpes simplex virus-2 (HSV-2) prevalence by age in men and women in fishing and inland communities. The solid line depicts the maximum a posteriori estimate of prevalence by age, the surrounding shaded area shows the 95% credible interval. The points represent the HSV-2 prevalence by age observed in the Sexually Transmitted Infection Prevalence Study (STIPS) data, vertical error bars show 95% binomial confidence intervals for each data point.

In results from the catalytic model (Supplementary Table 2), HSV-2 prevalence by age was markedly higher in women than in men and higher in the fishing community than inland (Figure 2). HSV-2 acquisition was younger for women than men in both inland and fishing communities. By age 20 years, 22.1% (95% credible interval [CrI], 15.3%–29.4%) of inland women had acquired HSV-2 and almost double that, 42.9% (95% CrI, 32.3%–53.5%), in the fishing community, compared to 11.3% (95% CrI, 5.5%–17.6%) and 10.7% (95% CrI, 4.1%–19.2%) of men in inland and fishing communities, respectively. By age 49 years, HSV-2 prevalence among women in the fishing community was 93.6% (95% CrI, 90.2%–96.6%) compared to 70.1% (95% CrI, 63.7%–76.8%) inland. In men, prevalence by age 49 years was 72.5% (95% CrI, 65.9%–79.5%) in the fishing community and 51.5% (95% CrI, 43.8%–59.9%) inland.

**Figure 3.**
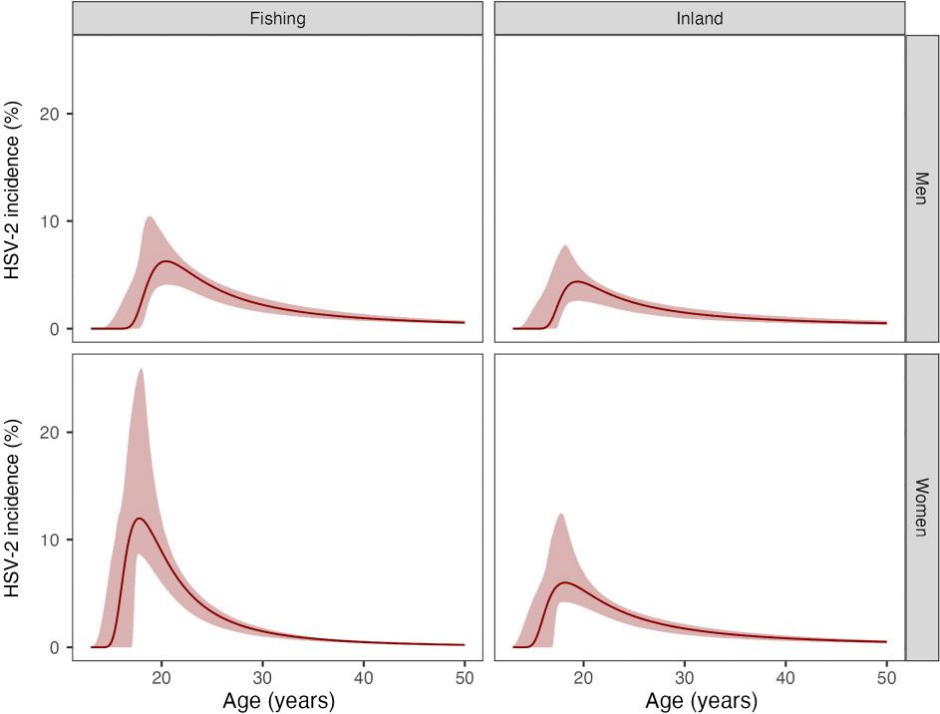
Modelled herpes simplex virus-2 (HSV-2) incidence per year by age in men and women in fishing and inland communities. The solid line depicts the maximum a posteriori estimate of prevalence by age, the surrounding shaded area shows the 95% credible interval.

Incidence of HSV-2 infection rose rapidly in late adolescence. The age pattern of infection was similar between women in both locations: annual incidence peaked at age 17.9 years (95% CrI, 16.5–19.0 years) in the fishing community and 18.1 years (95% CrI, 16.5–19.7 years) inland (Figure 3). However, annual incidence reached much higher levels in the fishing community: 15.6% (95% CrI, 8.6%–25.9%) compared to 7.3% (95% CrI, 4.2%–12.1%) inland. After the peak, annual incidence fell steeply to a similar level in both communities by age 30 years: 1.3% (95% CrI, 1.0%–1.8%) in the fishing community and 1.6% (95% CrI, 1.2%–2.2%) inland. The mean age of infection for women was 18.3 years in the fishing community and 21.0 years inland. Annual incidence in men peaked lower and at older ages compared to female peers, reaching 6.1% (95% CrI, 4.1%–8.5%) by age 20.3 years (95% CrI, 18.4–22.4 years) in the fishing community and 4.4% (95% CrI, 2.5%– 6.6%) by age 19.0 years (95% CrI, 17.0–21.4 years) inland. The mean age of HSV-2 infection for men was 21.1 years in the fishing community and 23.4 years inland.

## DISCUSSION

HSV-2 prevalence and incidence were extremely high in 2 distinct communities in south central Uganda, with marked heterogeneities by sex, community, and age. While the 2 communities studied were not selected to be representative of Uganda as a whole, the overall HSV-2 seroprevalence in our study was similar to national estimates from a recent meta-analysis (53.6%; 95% CrI, 51.3%–55.9% vs 50.5%; 95% CI, 44.2%–56.8%) [2]. Both estimates were notably higher than the 41.9% (95% CI, 38.4%–45.3%) prevalence estimated for the wider East Africa region. We found a clear difference in HSV-2 prevalence by sex, with women around 1.5 times more likely to have HSV-2. This ratio mirrored the meta-analysis findings sub-Saharan Africa, which estimated 43.1% (95% CI, 39.8%–46.5%) prevalence for women compared to 29.1% (95% CI, 25.7%–32.6%) for men.

Risk of acquiring HSV-2 infection peaked at very young ages, especially for women. HSV-2 prevalence was higher in women than in men in the same community at all ages, and we estimate incidence increased earlier. The mean age of infection was around 18 to 21 years in women and 21 to 23 years in men. The steep increase in incidence rates in late adolescence suggests that many people acquire infection soon after becoming sexually active; however, incidence rates remain high into early adulthood. Our findings are similar to those among women in Kilifi, Kenya, where Nyiro and colleagues observed “a rapid rise in HSV-2 seroprevalence from mid-late teenage years upwards, coincident with onset of sexual activity” [29]. However, they contrast with incidence patterns estimated in a meta-analysis of the whole sub-Saharan Africa region, where incidence was higher in those aged 25 years and older [2]. The age patterns of HSV-2 incidence were much younger than those seen for HIV in Rakai: the mean age of infection with HIV was 25 years for women and 29 years for men [30]. This age gap, combined with the increased risk of HIV seen among HSV-2–seropositive individuals, presents an important opportunity for prioritizing HIV prevention to a population with high HIV epidemic potential [31].

While the age profile of HSV-2 incidence was similar in the 2 communities studied, there were stark differences in the magnitude of the epidemics, with prevalence much higher in the fishing community than inland: 64% versus 43%. The higher burden of infection in the fishing community population aligns with previous findings on the extraordinarily high risk of STIs in this community, including 4 times greater prevalence of active syphilis than the national population [18].

At all ages, HIV prevalence was remarkably higher among those with HSV-2 than without, underscoring those with HSV-2 infection as a priority population for HIV prevention, either due to their position in sexual networks, of which HSV-2 serostatus is a marker, or increased risk of HIV acquisition associated with recent HSV-2 infection. Despite evidence that PrEP may offer partial protection against HSV-2 acquisition [32, 33], women with history of PrEP were more likely to be HSV-2 positive; although the association disappeared when the model was adjusted for confounders that may reflect underlying sexual risk, which could cause both higher HSV-2 prevalence and higher likelihood of PrEP eligibility. Furthermore, PrEP use within the cohort was quite low overall. While being circumcised was not significantly associated with reduced risk of HSV-2 after adjusting for confounders, interestingly the 25% point estimate of the reduction in risk was fairly close to the 28% found in a trial of voluntary medical male circumcision against HSV-2 acquisition [34].

The catalytic model has several key assumptions that should be considered when interpreting the results. Firstly, because it is not possible to distinguish age and cohort effects from cross-sectional data, our catalytic model implicitly assumes that age patterns of HSV-2 incidence have not changed over time. While it has been estimated that HSV-2 incidence in sub-Saharan Africa is declining at 2% annually due to the impact of HIV prevention [2], our estimate of 33.5% HSV-2 prevalence among inland men was essentially identical to that found in 2003, when 33.8% of men in the same community were found to be HSV-2 positive during enrolment for a randomized controlled trial [35]. Any population-level projections of future HSV-2 incidence trends should therefore consider the impact of cohort in addition to age. Secondly, stratified analysis by community type assumed that migration was not correlated with differential HSV-2 seroprevalence. Thirdly, we assumed that HSV-2 infection does not affect mortality. HSV-2 infection alone is unlikely to impact life expectancy, but its association with HIV may lead to higher mortality among HSV-2–positive individuals, biasing population HSV-2 prevalence at older ages. However, excess HIV mortality is now very low in the RCCS population, where 90% of people with HIV are virally suppressed [36]. Fourthly, we assumed there is no HSV-2 infection before people become sexually active, with the age of initiation of risk determined by model fitting in each population group. Although neonatal transmission of HSV-2 is an important public health concern associated with high infant mortality, it is estimated to occur in fewer than 2 in 10 000 live births across Africa [37], so population prevalence of infection in childhood is likely very low. The assumption of zero prevalence in childhood is therefore reasonable and unlikely to substantively impact the results.

Finally, our analysis is based on seroprevalence data from age 18 years onwards, slightly older than the peak age of incidence we inferred for women. Due to the lack of data informing the left tail of the age distribution of infection, we had to make informative prior assumptions about the minimum age of infection. Future studies collecting seroprevalence data down to age 15 years would provide greater resolution on patterns of HSV-2 infection at younger ages.

Despite estimates that the WHO Africa region has the highest HSV-2 burden globally, population-level studies of HSV-2 prevalence are sparse. Improved understanding of HSV-2 incidence patterns is vital to inform the design of intervention packages against HSV-2, which in turn could help reduce transmission of HIV by providing a marker of epidemic potential [31]. These results particularly inform the optimal age of delivery and which communities to prioritize. In the communities studied, HSV-2 infection likely occurs soon after commencement of sexual activity. Interventions against HSV-2 should therefore be targeted towards young people before they become sexually active. While there is not yet a vaccine against HSV-2, the WHO has identified the development of both therapeutic and prophylactic vaccines as an urgent priority [38]. In the meantime, interventions such as promoting condom use and voluntary medical male circumcision have been shown to reduce HSV-2 incidence [34]. Further synergies with HIV interventions may also be possible: building on earlier reports of in vitro activity of tenofovir against HSV-2, a study in serodiscordant heterosexual couples in Kenya and Uganda found that daily oral PrEP could reduce the risk of HSV-2 infection by 30% (95% CI, 1%–51%) [39]. We note that while the long-running HIV prevention program run by RHSP has achieved substantial reductions in HIV incidence with biomedical interventions [15], the lack of equivalent HSV-2 prevention measures has made reductions in infections harder to achieve, as evidenced by stable HSV-2 prevalence over 20 years [35]. In addition to quantifying underlying patterns of HSV-2 infection in 2 high-prevalence communities, our model provides a framework that can be applied more widely to improve our overall understanding of patterns of HSV-2 prevalence at a population level. Such estimates will be vital to design future interventions, such as vaccination.

## Supplementary Data

Supplementary materials are available at *The Journal of Infectious Diseases* online. Consisting of data provided by the authors to benefit the reader, the posted materials are not copyedited and are the sole responsibility of the authors, so questions or comments should be addressed to the corresponding author.

## Supplementary Material

jiad113_Supplementary_Data

## Notes

***Author contributions.*** The study was conceived by J. W. E., L. K. W., and M. K. G. The model was designed by J. W. E. and L. K. W. and implemented by L. K. W. Analysis was performed by L. K. W. and M. K. G. The data were originally collected by J. M., A. D. P., and R. S. M. Study funding was acquired by K. G., T. C. Q., and C. A. G. All authors had access to the data and accept responsibility for submission for publication. The report was drafted by L. K. W. and revised by all authors.

***Acknowledgments.*** We thank the personnel at the Office of Cyberinfrastructure and Computational Biology at the National Institute of Allergy and Infectious Diseases for data management support. Additionally, we thank the cohort participants and many staff and investigators who made this study possible.

***Disclaimer***. The views expressed are those of the authors and not necessarily those of the UK Department of Health and Social Care, NIH, NIHR, MRC, FCDO, UKHSA, or Wellcome Trust. The sponsors of the study had no role in study design, data collection, data analysis, data interpretation, or writing of the report. The corresponding author had full access to all the data in the study and had final responsibility for the decision to submit for publication.

***Financial support.*** This work was supported by the Center for AIDS Research, Johns Hopkins University (grant number P30AI094189); the National Institute of Allergy and Infectious Diseases (grant numbers R01AI143333, R01MH115799, and K01AI125086); the National Institute of Mental Health (grant number R01MH107275); the Eunice Kennedy Shriver National Institute of Child Health and Human Development (grant number RO1HD091003); the Division of Intramural Research of the National Institute for Allergy and Infectious Diseases, and the President’s Emergency Plan for AIDS Relief through the Centers for Disease Control and Prevention (grant number NU2GGH000817); the Wellcome Trust (grant number 218669/Z/19/Z to L. K. W.); and the MRC Centre for Global Infectious Disease Analysis jointly funded by the UK Medical Research Council (MRC) and the UK Foreign, Commonwealth, and Development Office (FCDO), under the MRC/FCDO Concordat agreement, and the EDCTP2 program supported by the European Union (grant number MR/R015600/1 to L. K. W. and J. E.). Y. C. M. receives salary support from the National Institutes of Health (grant numbers U54EB007958 and D43TW009771). Funding to pay the Open Access publication charges for this article was provided by the Wellcome Trust.

***Potential conflicts of interest.*** Y. C. M. has received grant support to John Hopkins University from Hologic, Cepheid, Roche, ChemBio, Becton Dickinson, and miDiagnostics, and has provided consultative support to Abbot. All other authors report no potential conflicts.

***Data sharing.*** All data and code are available upon reasonable request to the Rakai Health Sciences Program.
