# Supplementary material for: Age Patterns of HSV-2 Incidence and Prevalence in Two Ugandan Communities: A Catalytic Incidence Model Applied to Population-Based Seroprevalence Data
Source: J Infect Dis. 2023 Apr 20;228(9):1198–207. doi: 10.1093/infdis/jiad113 (PMC10629710; doi:10.1093/infdis/jiad113)
Supplement: jiad113_Supplementary_Data [file jiad113_supplementary_data.docx]

Supplementary Material

# 1. Supplementary Methods

We represented prevalence at age $x$ using the cumulative distribution function of shifted generalised gamma distribution, a flexible distributional form which incorporates the exponential, gamma, Weibull, and lognormal distributions as special cases (Prentice, 1974; Cox *et al.*, 2007). Prevalence of HSV-2 infection at age $x$ was therefore:

$\begin{aligned} F\left( x| \mu, \sigma,q,x_{0} \right)=\frac{\gamma\left( k,kw \right)}{\Gamma\left( k \right)} \#(1) \end{aligned}$where

$$w=\frac{\log\left( x-x_{0} \right)-\mu}{\sigma}, k=\frac{1}{q^{2}}$$

and $\Gamma\left( \cdot\right)$ and $\gamma\left( \cdot, \cdot\right)$ represent the gamma and lower incomplete gamma functions, respectively. The model parameters can be interpreted as representing the location ($\mu)$, scale ($\sigma)$ and shape ($q)$ of the generalised gamma distribution, with an additional ‘shift’ parameter $x_{0}$ representing the minimum age of HSV-2 infection.

Since infection with HSV-2 is lifelong, the prevalence of infection by age is equivalent to cumulative incidence of infection $F\left( x \right)=\int_{0}^{x} f\left( z \right)dz$. The incidence rate at age $x$ is the probability density function of the shifted generalised gamma distribution.

$$\begin{aligned} f\left( x;\mu, \sigma,q,x_{0} \right)=\frac{\left| q \right|}{\sigma\left( x-x_{0} \right)\Gamma\left( k \right)}k^{k}{\exp\left\{ k\left( qw-e^{qw} \right) \right\}} \#\left( 2 \right) \end{aligned}$$

where $\mu, \sigma>0$. We restricted the shape parameter $q<0$, to ensure the incidence curve had positive skew (2).

We used a binomial distribution to represent the likelihood $L(Y)$ of observing $y\left( x \right)$ HSV-2 infections out of $N\left( x \right)$tests. The probability of observing a positive test at age $x$ is the population prevalence at that age, $F(x)$:

$$L\left( Y|N, \mu, \sigma, q, x_{0} \right)=\prod_{x=18}^{49} \binom{N\left( x \right)}{y\left( x \right)}F\left( x \right)^{y\left( x \right)}\left( 1-F\left( x \right) \right)^{N\left( x \right)-y(x)}$$

We adopted diffuse improper uniform priors for the location, scale, and shape parameters, such that $\mu,\sigma\sim U\left[ 0, \infty\right);q\sim U(-\infty,0]$. For the shift parameter we assumed a gamma prior corresponding to a 95% probability that the minimum age of HSV-2 infection is in the interval 12-16.5, i.e., $x_{0}\sim\text{Gamma}(150, 10.6)$.

We implemented the model in a Bayesian framework using the probabilistic programming language Stan (3). We used an adaptive Hamiltonian Monte Carlo (HMC) no U-turn sampler to estimate model parameters for each location and gender separately. For each fit, four parallel chains of the HMC were run for 10,000 iterations each, with the first 5,000 discarded as burn-in. We assessed convergence by ensuring the multivariate Gelman-Rubin (GR) diagnostic was <1.1 for all parameters (4) and the effective sample size (ESS) for the combined chains was >1,000. Analyses were performed using RStan version 2.21.3 within R version 4.0.2.

We calculated the age of peak HSV-2 incidence under each sampled parameter set by deriving expressions for the mean and mode of the shifted generalised gamma distribution:

$$\begin{aligned} x_{mode}= x_{0}+e^{\mu}\left( 1-\sigma q \right)^{\frac{\sigma}{q}}\#(3) \end{aligned}$$

$$x_{mean}=x_{0}+e^{\mu}k^{\frac{\sigma}{q}}\frac{\Gamma\left( k\left( 1-\sigma q \right) \right)}{\Gamma(k)}$$

## 2. Supplementary Results

Table S1 Associations between curable sexually transmitting infections and HSV-2

|  | **Women (N=960)*** | | | | **Men (N=859)*** | | | |
| --- | --- | --- | --- | --- | --- | --- | --- | --- |
|  | **No. HSV-2 seropositive/Total** | **%** | **PRR (95%CI)** | **adjPRR (95%CI)** | **No. HSV-2 seropositive/Total** | **%** | **PRR (95%CI)** | **adjPRR (95%CI)** |
| **Chlamydia** |  |  |  |  |  |  |  |  |
| No | 545/761 | 63 | Ref. | Ref. | 342/771 | 70 | Ref. | Ref. |
| Yes | 59/97 | 61 | 0.96 (0.81-1.14) | 1.14 (0.97-1.33) | 24/80 | 30 | 0.68 (0.48-0.95) | 0.83 (0.61-1.14) |
| **Gonorrhea** |  |  |  |  |  |  |  |  |
| No | 553/883 | 63 | Ref. | Ref. | 342/805 | 42 | Ref. | Ref. |
| Yes | 51/75 | 68 | 1.09 (0.92-1.28) | 1.04 (0.91-1.20) | 24/46 | 52 | 1.23 (0.92-1.64) | 1.06 (0.83-1.35) |
| **Trichomonas** |  |  |  |  |  |  |  |  |
| No | 481/808 | 60 | Ref. | Ref. | 344/808 | 43 | Ref. | Ref. |
| Yes | 125/152 | 82 | 1.38 (1.26-1.52) | **1.22 (1.12-1.34)** | 22/43 | 51 | 1.20 (0.89-1.63) | 0.95 (0.71-1.28) |
| **Positive syphilis serology** |  |  |  |  |  |  |  |  |
| No | 482/809 | 60 | Ref. | Ref. | 276/727 | 30 | Ref. | Ref. |
| Yes | 124/151 | 82 | 1.38 (1.26-1.51) | 1.05 (0.96-1.15) | 93/132 | 71 | 1.86 (1.61-2.14) | **1.26 (1.08-1.46)** |
| **High-titer syphilis** |  |  |  |  |  |  |  |  |
| No | 569/914 | 62 | Ref. | Ref. | 334/807 | 41 | Ref. | Ref. |
| Yes | 37/96 | 80 | 1.29 (1.11-1.50) | 1.07 (0.91-1.26) | 35/52 | 67 | 1.63 (1.33-2.00) | 1.14 (0.93-1.39) |
| PRR=Prevalence Risk Ratio; adjPRR=adjusted prevalence risk ratio; models adjusted for age, community type, marital status, educational level, and HIV serostatus. *There were six study participants with missing HSV-2 results (n=5 women; n=1 man) | | | | | | | | |

Table S2 Posterior parameter estimates, mean (95% Credible Intervals)

|  |  | **Inland** | | **Fishing** | |
| --- | --- | --- | --- | --- | --- |
| **Parameter** | **Description** | **Women** | **Men** | **Women** | **Men** |
| $\mu$ | Location | 1.8 (0.9, 2.6) | 2.0 (0.9, 3.1) | 1.5 (0.8, 2.0) | 2.1 (1.4, 2.7) |
| $\sigma$ | Scale | 0.8 (0.1, 1.6) | 0.8 (0.1, 1.9) | 0.5 (0.1, 1.1) | 0.7 (0.1, 1.2) |
| $q$ | Shape | -4.5 (-21.4, -0.4) | -6.5 (-27.4, -0.5) | -3.6 (-14.7, -0.3) | -2.9 (-11.8, -0.4) |
| $x_{0}$ | Shift | 14.2 (12.1, 16.4) | 14.4 (12.2, 16.7) | 14.2 (12.1, 16.4) | 14.3 (12.1, 16.6) |
| $x_{mode}$ | Age of peak incidence | 18.1 (16.5, 19.7) | 19.0 (17.0, 21.4) | 17.9 (16.5, 19.0) | 20.3 (18.4, 22.4) |
| $x_{mean}$ | Mean age of infection | 21.0 (14.5, 37.8) | 23.4 (14.2, 60.6) | 18.3 (15.5, 22.4) | 21.1 (15.5, 33.2) |

# Supplementary references

1. Cox C, Chu H, Schneider MF, Munoz A. Parametric survival analysis and taxonomy of hazard functions for the generalized gamma distribution. Statistics in Medicine. 2007;26:4352–74.

2. Prentice RL. A log gamma model and its maximum likelihood estimation. Biometrika. 1974;61(3):539–44.

3. Betancourt M. A Conceptual Introduction to Hamiltonian Monte Carlo. 2017; Available from: http://arxiv.org/abs/1701.02434

4. Brooks SPB, Gelman AG. General methods for monitoring convergence of iterative simulations. Journal of Computational and Graphical Statistics. 1998;7(4):434–55.
